# 

**Published:** 1843-08-19

**Authors:** 


### CONTENTS.

Remarks on Uterine Polypus, with a Case of Extirpation of the same by the knife. By John M. Turner, M. D. - 181

*Retrospect of the Medical Sciences.*

Dr. M'Cormac on Prolapsus Ani, - 184

Employment of Restraint in the Treatment of Insanity, - - - - 184

A few observations on Encysted Hydrocele. By Robert Liston, F. R S. - 185

Effects of Menstruation on the Secretion of Milk, - - - - - - - 185

Antidote for the Bichloride of Mercury, 186

Sir Henry Marsh on Regurgitation, - 186

Mr. Hamerton on Acute Glanders in the Human subject, - - - - 186

Dr. Sutherland on the Blood in Insanity, 187

Hemiplegia from tying the common Carotid Artery, - - - - - - 188

Dr. Wilson on Nervous Disorders, - 189

Influence of External Agencies on Disease, - - - - - - - 191

Spontaneous cure of Ovarian Dropsy, - 192

New mode of administering Mercury, and theory of its changes in the system, 192

Statistics of Fractures, - - - 192

